# Value of Platelet to Lymphocyte Ratio in Nasopharyngeal Carcinoma Various Stages

**DOI:** 10.22038/IJORL.2024.75070.3523

**Published:** 2024-05

**Authors:** Goesti Yudistira, Melati Sudiro, Yussy Afriani Dewi

**Affiliations:** 1 *Department of Otorhinolaryngology-Head and Neck Surgery, Faculty of Medicine Padjadjaran University/ Dr. Hasan Sadikin General Hospital Bandung, Indonesia. *

**Keywords:** Lymphocytes, Nasopharyngeal Carcinoma, Platelet Count, Plaletets-to-Lymphocyte Ratio, Prognosis

## Abstract

**Introduction:**

Nasopharyngeal Carcinoma (NPC) is among the most common head and neck cancer, significantly affecting the growth of human tumors. Therefore, this study aimed to investigate platelet-to-lymphocyte ratio (PLR) as a measure of inflammation in patients with various stages of NPC.

**Materials and Methods:**

This retrospective case-control study was conducted on patients diagnosed with NPC between 2016 and 2020 in Dr. Hasan Sadikin Bandung Hospital, Indonesia. Based on NPC classification criteria, patients were classified into early and advanced groups. Subsequently, the PLR ratio was calculated as the number of platelet divided by the absolute number of lymphocytes in the two groups, and the results were statistically analyzed using Unpaired T-test, Pearson, and Chi-square test.

**Results:**

A total of 102 patients with an age range of 40 to 49 years were included in this study. A slight correlation of 0.272 (P<0.001) was observed between PLR and clinical stage, while NPC patients with PLR value > 188 had a 2.7 times higher risk of advanced stage (RR=2.702).

**Conclusion:**

The PLR was significantly related to the clinical stage of NPC cancer.

## Introduction

Nasopharyngeal Carcinoma (NPC) is a malignant tumor originating from nasopharyngeal epithelium, serving as the predominant head and neck malignancy in Southeast Asia. This malignancy shows a predilection for the lateral wall of the nasopharynx, also known as the Rosenmüller Fossa (1–3).

For decades, NPC has been endemic across several regions including in the Arctic, East, and Southeast Asia, as well as the Middle East, and North Africa. 

The International Agency for Research on Cancer estimates that the prevalence of NPC in Asia is 85.2%, with an incidence rate of 6.2/100.000 people in Indonesia. At the Department of Health Ear Nose Throat Head and Neck Surgery (T.H.T.K.L) in Dr. Hasan Sadikin Bandung Hospital (RSHS), NPC is regarded as the most common head and neck malignancy with 921 new cases, comprising 67% male (4–7).

Nasopharyngeal biopsy is used to diagnose NPC, including physical, laboratory, and radiological examinations. The clinical symptoms include epistaxis, nasal congestion, tinnitus, otalgia, diplopia, ear fullness, abnormalities of the cranial nerves (III, IV, V, and VI), and lump in the neck showing advanced stage (4,6).

Inflammation plays an important role in the development of tumors and angiogenesis. This is because tumor-associated response is composed of inflammatory mediators and cells. Therefore, inflammation-based tests are developed to predict oncological outcomes in different solid tumors including the Neutrophil-to-Lymphocyte Ratio (NLR), Creative Protein-to-Albumin Ratio (CAR), and Platelet-to-Lymphocyte Ratio (PLR) (8–10). 

Several studies have shown that platelet contribute to the production of inflammatory cytokines and chemokines, supporting angiogenesis and stromal development by secreting vascular endothelial factors. Meanwhile, lymphocytes significantly contribute to the immunological anticancer response through the inhibition of tumor proliferation and induction of cell death. This immune response includes tumor-infiltrating lymphocytes which are useful in several stages of tumor formation. The PLR is a representative of promoting tumor functions and anti-tumor immune reactions in NPC to monitor the development of tumors. Therefore, this study aims to explore the relationship between PLR and NPC at the clinical stage (11–13).

## Materials and Methods

This retrospective case-control analytical study was conducted in Dr. Hasan Sadikin Bandung General Hospital with stratified random sampling methods. Data were collected from 2016 to 2020 at Nasopharyngeal Cancer Register System for the Oncology Head and Neck Surgery Study Group, Indonesian Otorhinolaryngology-Head and Neck Surgery Society (PERHATI-KL). Subsequently, the PLR was calculated by platelet count divided by absolute lymphocyte count.

In this study, the inclusion criteria were patients with NPC diagnoses, no prior cancer treatment, and full medical record data. Meanwhile, the exclusion criteria were primary malignancy, recurrent NPC, a history of diabetes mellitus, or cardiovascular disease. The characteristic of the participants was presented descriptively, while the Chi-Square test was used to analyze the relationship between the PLR and NPC clinical stage. Additionally, the statistical test was established with a (P<0.05), followed by data analysis and processing using SPSS Version 24.0 software.

## Results

Among 588 NPC patients observed from January 2016 to December 2020, 383 met the inclusion criteria for this study. The participants were divided into early and advanced groups, consisting of 51 each selected using stratified random sampling. 

Table 1 shows the characteristics of gender, age, TNM classification, and clinical stage. Based on the results, approximately 65.7% of the participants were male, with 40.2% aged 40–49 years, while the youngest was 16 years old and the oldest was 77 years old. According to the AJCC 8th TNM classification, the highest percentages were observed in T2, N0, and M0 at 43.13%, 33.3%, and 87.2%, respectively. The early clinical stage was found to be stage I (8.8%) and II (41.2%), while advanced stage consisted of III, IVA, and IVB, with proportions of 16.6%, 20.7%, and 12.7%, respectively.

**Table 1:** Demographic characteristics

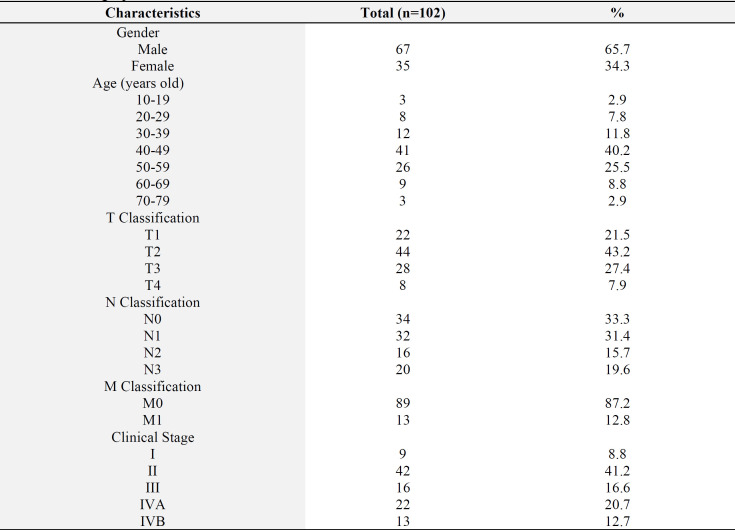

Table 2 shows that PLR value at the early and advanced stage have significant differences with a correlation coefficient of p=0.006 and r=0.272. 

**Table 2:** Correlation of the PLR with Clinical Stage

**PLR**	**Clinical Stage**		**pa**	**Correlation Coefb**
	**Early**	**Advanced**		
Mean	185,8	290,8	0.006	0.272
Range	45-1074	52-1473		

Note: aUnpaired T-test,bPearson test

**Fig 1:**
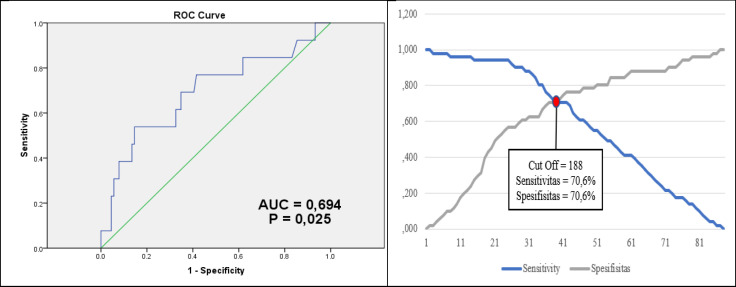
ROC Curve for the PLR Cut-Off

Figure 1 shows that the PLR cut-off value was determined using the ROC curve. Based on the results, the cut-off of PLR value was 188 with P=0.025, an AUC of 0.694 with 60.6% sensitivity and 70.6% specificity. 

Table 3 shows that the clinical stage of NPC and PLR value had a significant connection of p<0.001. Furthermore, NPC patients with PLR value exceeding 188 were 2.7 times higher at an advanced stage with PLR ≤ 188.

**Table 3:** Correlation of the PLR with Clinical Stage

**PLR**	**Clinical Stage**		p*	**RR (95%CI)**	
	**Advanced (n=51)**	**Early (n=51)**		
>188 ≤188	38 (37.3%) 13 (12.7%)	15 (14.7%) 36 (35.3%)	<0.001	2.702 (1.646-4.436)	

Note: *Chi-Square

## Discussion

NPC ranks fourth among head and neck malignancies with an incidence rate of 85.2% in East and Southeast Asia, where China has the highest number of cases, followed by Indonesia (14,15). 

However, there is a decrease in NPC incidence in Hong Kong, Taiwan, and Singapore due to the shift from the traditional Chinese to Western diet patterns because of economic growth. This shows that nutrition plays a significant role in NPC pathophysiology. Based on the case study, the dietary habits of Asians who consume smoked meat, salted fish, aged vegetables, preserved vegetables, and drinking herbal teas were related to a higher risk of NPC. The increase in cases among youth in South China was related to the early ingestion of salted fish. According to Barrett *et al.,* the consumption of salted fish only increased the incidence of NPC in childhood and not in adolescents or adults (15,16).

Based on the analysis conducted in this study, ratio of male to female participants was found to be 1.9:1. This corresponds to the reports by WHO, where male has a higher incidence of NPS compared to female in Southeast Asia with ratio of 3:1. A similar study conducted by Hardianti *et al*., stated that male had more cases with ratio 1.7:1 at Hasan Sadikin Hospital. This ratio is higher in male affected by drinking alcohol and smoking, which increase NPC risk by 5.8 and 3.6 times, respectively. Meanwhile, the presence of these two lifestyles increased the risk of NPC by 19 times (14,17).

Li *et al*. discovered that ratio of male NPC patients who smoked and drank alcohol was 70 times higher. According to Long *et al*, smoking increases the risk of developing NPC later in life of female (15,18,19). This study also showed that the 10-year survival rate of female NPC patients was higher than males Zuo *et al*. showed that people are susceptible to NPC because the male X chromosome contains the Human Leucocyte Antigen (HLA), namely chromosome 6, which is vulnerable to NPC. Meanwhile, postmenopausal survival rates are lower in female with NPC because estrogen levels which serve as a defense mechanism are lower after menopause (20,21).

A crucial element in NPC pathophysiology is the patients’ DNA, specifically the telomeres, which are the ends of DNA chain filled with proteins. The telomeres length in NPC patients is 3.2 times shorter than normal, which contributes to the development of tumors. According to Ko *et al*., telomeres shortening is related to aging and the male gender, serving as predictive indicators for poor prognosis in NPC patients (22).

In NPC, inflammation contributes significantly to leukocytosis, thrombocytosis, neutrophilia, and lymphocytopenia. Recent studies suggest that inflammatory responses contribute to the initiation, progression, and prognosis of numerous cancers. This shows that inflammatory-based hematological examination of cancer is useful, playing an important role in tumor proliferation and metastasis. Immune cells and inflammatory proteins that infiltrate tumors also show the connection between hematologic characteristics and cancer, although the exact mechanism is unknown. Several immune cells that influence the malignancy phenotype include lymphocytes, endothelial, mesenchymal, inflammatory mediators, and extracellular matrix molecules (23,24).

Lymphocytes contribute to cancer immune surveillance and prevent the development of malignancy. In several cancer forms, elevated TILs have shown a significant correlation to a favorable outcome. Yu Tzu Huang *et al.* discovered that the primary cytokine components isolated from nasopharyngeal biopsy preparations were lymphocytes CD4+ and CD8+. According to Barele *et al*., TILs in NPC patients were connected to increased Overall Survival (OS) (23,25). 

Antigens contribute significantly to the initiation of immune responses in NPC against cancer cells. 

These antigens are expressed with MHC class 1 by producing TCR and lymphocytes T CD8+ to become active as Tc lymphocytes, which eliminate tumors and transform into memory cells. Additionally, lymphocytes T CD4+ become active as Th lymphocytes, express IFN-ɣ, and TNF-α, which increase the MHC class I molecule and improve the cytotoxic function of Tc lymphocytes. 

A small portion of antigens is expressed with MHC class II and forms a complex with lymphocytes T CD4+. The cross-presentation mechanism causes CD4+ Th cells to simulate Tc lymphocytes in eradicating tumor cells (26,27).The production of cytokines IL-1, IL-6, Granulocyte-Macrophage Colony-Stimulating Factor (GM-CSF), and Granulocyte-Colony-Stimulating Factor (G-CSF), which induce thrombopoiesis as well as megakaryopoiesis, cause thrombocytosis in cancer patients. The membrane glycoproteins interacting with tumor cells and attachment of P-selectin also serve as mediators for platelet protection from cancer (8,28). 

Platelet also prevents natural killer cells (NKC) from recognizing cancer cells by activating TGF-β and Nuclear Factor-kB (NF-kB). Additionally, platelet transfer "normal" MHC class I molecules by preventing from being recognized as foreign cells, interfering with the ability of NKC, which is decreased by TGF-β to produce cytotoxicity and IFN- γ. This phenomenon indirectly reduces or eliminates the ability of lymphocytes T CD8+ to detect cancer cells (28).

Cancer cells produce IL-6 and thrombopoietin to exploit protumor impact and induce paraneoplastic thrombocytosis. ADP and thrombin molecules are released to activate platelets, which worsens the outcomes for cancer patients. Consequently, platelet count can be a prognostic indicator for NPC (29).PLR, combining platelet and lymphocyte counts, is a representative index of systemic inflammation and immunological status, which can be used as a prognostic and diagnostic marker in several types of cancers. Ye *et al*. reported that the average PLR value in healthy people was 105, while the NPC group was 122 with a range from 40 to 253 (24,30).

In gastric cancer, the diagnostic value of PLR was found to be higher than the traditional tumor markers CEA (Carcinoembryonic Antigen) and CA19-9 (Carbohydrate Antigen 19-9). An elevated pre-treatment PLR was identified as a prognostic factor for poor OS and DFS, along with poor clinicopathological parameters in gastric cancer (24,31). 

Prognostic value of preoperative PLR was discovered by Tu *et al. *in patients with laryngeal squamous cell carcinoma (LSCC), where high PLR (>114) were more frequent in LSCC with advanced T stage. Additionally, value of PLR is significantly associated with tumor size (T) in NPC (8,32). 

Aksimitayani *et al.* found that PLR was used as a post-chemotherapy evaluation. After three cycles of chemotherapy, a decrease was observed in PLR due to side effects of bone marrow suppression experienced by patients with paclitaxel-cisplatin regimen. Paclitaxel is attached to microtubules to prevent depolymerization and the mitosis of hematopoietic cells as well as harm the mitochondrial DNA. 

Furthermore, cisplatin is attached to bone marrow and damages it during DNA replication, causing a significant decrease in platelets and lymphocytes in the bloodstream (33). Based on the results, NPC patients with PLR value exceeding 188 had a 2.7 times risk of progressing to advanced stage. According to Peng *et al*., NPC patients with PLR value >157 had poor OS and PFS (34).

The limitations of this retrospective study included the use of a relatively small size and a single center for the experiment, although covering clinical stages I through IVB. Furthermore, the existence of comorbidities in certain cases led to uncertainty due to the correlation between the two aspects of the immune system and other diseases. For further studies related to randomized controlled trials (RCTs), an appropriate cut-off PLR is required to predict the risks of advanced stage and survival outcome.

## Conclusion

In conclusion, this study showed that elevated PLR (>188) was significantly related to advanced stage of NPC. The results suggested that PLR could serve as a cost-efficient method, providing reliable information before treatment for NPC patients. Moreover, further studies are recommended to clarify the exact mechanisms and function of PLR on NPC. 
